# A conceptual approach to material detection based on damping vibration-force signals via robot

**DOI:** 10.3389/fnbot.2025.1503398

**Published:** 2025-02-11

**Authors:** Ahmad Saleh Asheghabadi, Mohammad Keymanesh, Saeed Bahrami Moqadam, Jing Xu

**Affiliations:** ^1^State Key Laboratory of Tribology, The Beijing Key Laboratory of Precision/Ultra-Precision Manufacturing Equipment Control, The Department of Mechanical Engineering, Tsinghua University, Beijing, China; ^2^State Key Laboratory of Tribology in Advanced Equipment, Department of Mechanical Engineering, Tsinghua University, Beijing, China; ^3^Department of Control Science and Engineering, Tongji University, Shanghai, China; ^4^National Key Laboratory of Autonomous Intelligent Unmanned Systems, Shanghai, China; ^5^Frontiers Science Center for Intelligent Autonomous Systems, Shanghai, China

**Keywords:** cantilever beam mechanism, damping force signal and damping vibration, material detection, vibration amplitude, damping time, wavelength, cantilever beam's deflection

## Abstract

**Introduction:**

Object perception, particularly material detection, is predominantly performed through texture recognition, which presents significant limitations. These methods are insufficient to distinguish between different materials with similar surface roughness, and noise caused by tactile movements affects the system performance.

**Methods:**

This paper presents a straightforward, impact-based approach to identifying materials, utilizing the cantilever beam mechanism in the UR5e robot's artificial finger. To detect object material, an elastic metal sheet was fixed to a load cell with an accelerometer and a metal appendage positioned above and below its free end, respectively. After recording the damping force signal and vibration data from the load cell and accelerometer caused by the metal appendage's impact, features such as vibration amplitude, damping time, wavelength, and force amplitude were retrieved. Three machine-learning techniques were then used to classify the objects' materials according to their damping rates. Data clustering was performed using the deflection of the cantilever beam to boost classification accuracy.

**Results and discussion:**

Online object materials detection shows an accuracy of 95.46% in a study of ten objects [metals (steel, cast iron), plastics (foam, compressed plastic), wood, silicon, rubber, leather, brick and cartoon]. This method overcomes the limitations of the tactile approach and has the potential to be used in industrial robots.

## 1 Introduction

Over several decades, robots have replaced humans for greater efficiency and manipulation of objects in various tasks in diverse environments, such as industrial automation, repetitive tasks, and social interaction/assistance. Hence, significant steps have been taken to enhance the skills of robots and bring them closer to human capabilities (Wei et al., 2023; Chuang, 2024). Robots are typically programmed with the capability to replicate intricate and multifaceted tasks. To achieve this, researchers define six important indicators, including kinematic architecture, activation, transmission, sensing, materials, and construction to enhance their functional dexterity (Controzzi et al., 2014). In line with sensing, most research efforts have focused on developing surface artificial tactile sensors that provide sufficient information for dexterous manipulation. This development includes the integration of sensors for detecting materials' characteristics, such as object edge shape (Suwanratchatamanee et al., 2007), surface properties like hardness and texture (Johnsson and Balkenius, 2008; Bok et al., 2021), and material discrimination (Lee et al., 2021). These sensors use capacitive (Tavakoli et al., 2017), piezoresistive (Fonseca et al., 2023; Kappassov et al., 2015), optical (Jiang et al., 2015), and magnetic (Kim et al., 2018) technologies, all of which are activated by mechanical stimulation. The ultimate aim is to equip robots with complex tactile perceptions similar to those of humans (Jamali and Sammut, 2011).

To assist robots in dexterous manipulation, embedded sensors must receive sufficient information from the target to effectively interact with their surroundings. For material detection, the proposed methods usually rely on sensor rubbing/friction and acoustic-based tapping on objects (Okamura et al., 1997; Spiers et al., 2016). For instance, Jamali and Sammut (2011) proposed an artificial silicon finger consisting of strain gauges and polyvinylidene fluoride (PVDF) films and embedding it into the gripper, which was able to classify eight object materials based on their texture. Friction between the sensor and different object textures induced vibrations in the silicon. Then, a classification accuracy of 95% was achieved using a Naive-Bayes tree (NBTree) classifier by extracting different frequencies. Shuaikang et al. (2023) embedded two force sensors at the fingertips (index and thumb) of the RH8D five-fingered robotic hand to measure object recognition based on hardness and texture through the force generated via rubbing the fingertips against objects. They classified seven materials via the support vector machine (SVM) algorithm using the Fourier transform (FFT) features with an accuracy of 86%. Tanaka et al. (2019) presented an artificial finger with different horizontal ridges to evaluate the effect of varying roughness on the vibration sensor output. The researchers rubbed nine objects of varying roughness with an approximate force of 0.3 N. The results indicated that various artificial fingers have different responses, reflecting differences in horizontal ridges. In 1996, Krutkov et al. introduced a method for finding materials based on their acoustic properties (Krotkov et al., 1997). To sort four materials into groups, they tapped the robot's end-effector into objects. Subsequently, other researchers presented alternative methods. Sinapov et al. (2009) used a Barrett WAM robot to create airborne sounds by performing tapping as an exploratory action on various objects. Their objective was to classify 36 different household objects using a self-organizing map (SOM), k-nearest neighbor (KNN), and support vector machines (SVM). The results showed that it is possible to identify these objects with an average accuracy of around 73%. The authors used a robotic system that implemented a latent regularized variational autoencoder (DLR-VAE) in Neumann et al. (2018). They recorded data by knocking on objects and grasping them. The encoder then mapped the input data into the latent space using a multiple-layer perceptron (MLP). Next, the features were extracted by transferring from the time domain to the frequency domain, resulting in the classification of eight materials with 76% accuracy.

However, the sensors introduced are difficult to replicate or usually bulky to achieve precise spatial resolution. On the other hand, commercial sensors, while providing good spatial resolution, only respond to stimuli that are normal to the sensor surface. To reduce wiring complexity, they use scanning techniques for data acquisition, thereby increasing the cost. Furthermore, the thickness and strength of the ridges influence mechanical stimulation. Therefore, it relies on tactile parameters that must be tailored to suit various applications (Agache et al., 1980). Furthermore, because acoustic-based material recognition necessitates the use of microphones, it has received less research than tactile-based material recognition, as soft materials don't generate sound that the microphone can record.

Although the presented methods have increased the performance of robots, the noise caused by tactile movements affects the system's performance (Chen et al., 2019). On the other hand, these methods rely on applying additional mechanical energy to enhance the perception of surface roughness (Hendriks and Franklin, 2010). Furthermore, texture-based material discrimination methods are not sufficient to distinguish between different materials with similar surface roughness. Consequently, object material recognition remains an open challenge. Hence, the primary motivation is to eliminate the influence of texture and material classification with a similar surface texture via an artificial finger that can be mounted on a robot. The novelty of our research is the approach in which, rather than using tactile methods to identify materials, we use the approach of damping signals by impacting the objects with a finger to acquire the signals. Our approach is based on a cantilever beam mechanism in which a load cell and an accelerometer are embedded in a cantilever beam made of an elastic metal sheet. Furthermore, we fixed a rigid metal appendage to impact the object at its free end. This setup allowed for simultaneous recording of the damping vibration-force signals when the metal appendage impacts with objects. The collected signals were then used as input for the classification model. Additionally, we used a clustering technique to reduce the machine's input data, thereby improving system performance. The presented approach allows machine learning to make an effective association between the robot and objects.

To summarize, the main contributions of this paper are as follows: (a) Reducing noise and signal interference compared to traditional tactile methods through damped vibration signals, (b) We present a straightforward method for detecting intrinsic material properties using a cantilever beam, removing the need for surface features. This design choice makes the system less sensitive to variations in roughness and more reliable in distinguishing similar materials, (c) Reducing the amount of data processed through clustering improves the system's efficiency, lowering computational load and increasing the robot's response speed.

The following section gives an overview of the remaining work. Section 2 presents the methodology. Section 3 describes the experiments and results. Then, we detail the discussion in Section 4. The paper concludes with Section 5.

## 2 Method

**Figure 1:**
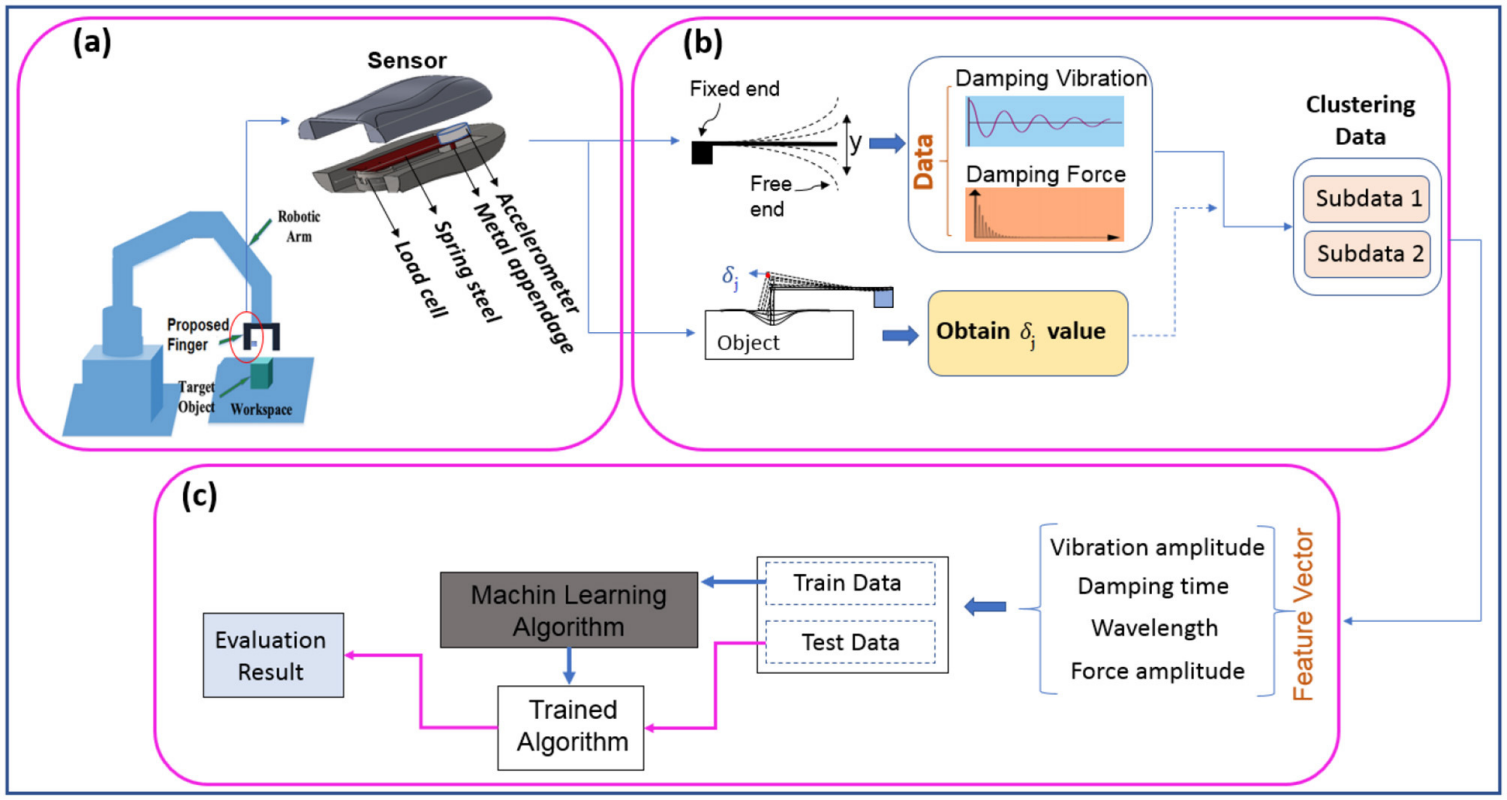
Overview of presented method from receiving signal to object material detection: **(A)** schematic of the sensor embedded in the robot, **(B)** acquisition of vibration-force damping signals by impact approach and clustering using beam deflection value, **(C)** classification of material based on extracted features.

Figure 1 describes the flowchart of the material detection of the objects with the proposed sensor via the UR5e robot, including: (a) A schematic of the proposed sensor embedded in the UR5e robot. (b) Acquisition of damping vibration-force signals through an impact approach and then clustering them using the beam deflection value. (c) Material classification based on the extracted features. Each section will be detailed separately.

### 2.1 Sensor design

**Figure 2:**
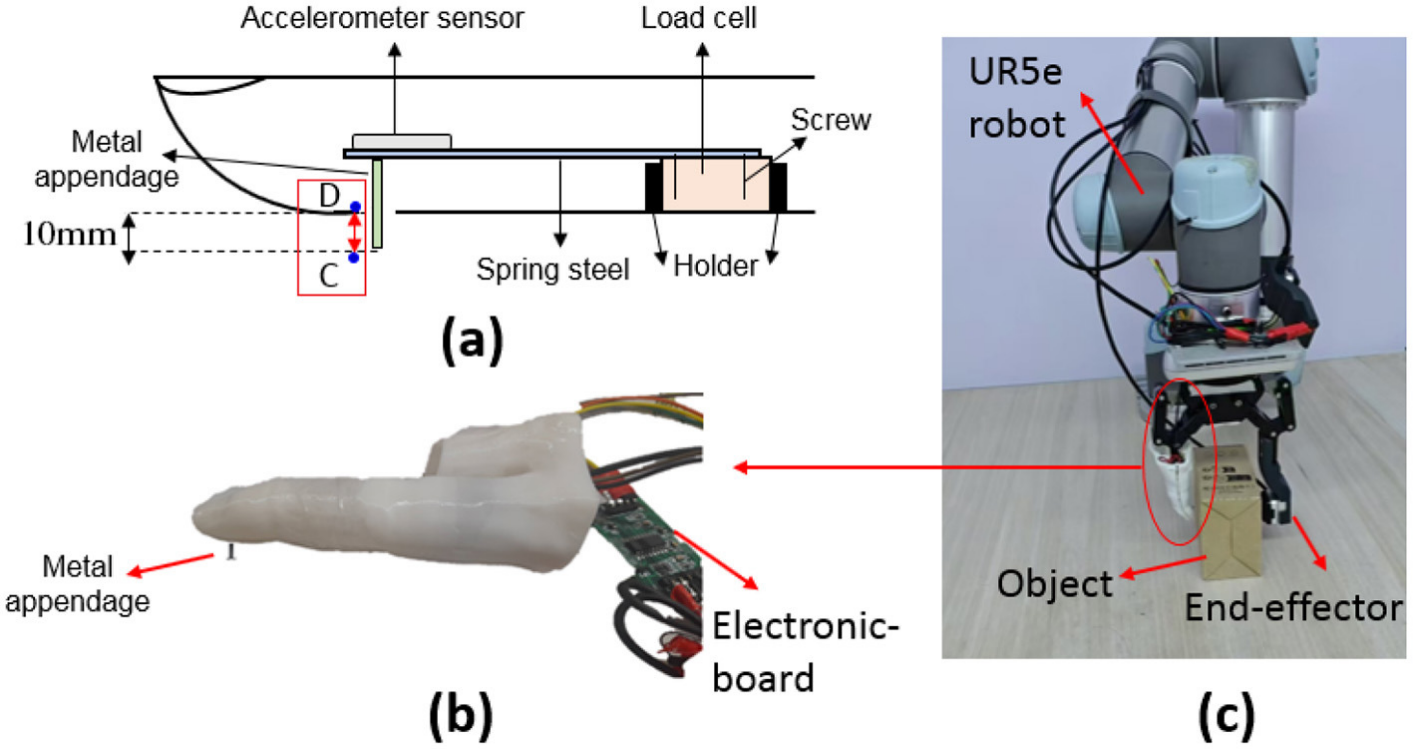
Experimental setup **(A)** schematics and embedded components of the sensor. **(B)** 3D printing artificial finger. **(C)** Artificial finger mounted on a UR5e Robot gripper.

The proposed material detection sensor is shown in Figure 1A. It is made up of a 20-mm-wide spring metal sheet that acts as a cantilever beam, with one end attached to a DYZ-100 miniature tensile strain sensor that is small, light, and accurate. The load cell converts the force into a measurable electrical output with an accuracy of 0.03% to 0.25%. Also, an accelerometer (13-bit resolution) is placed on its free end. This sensor was embedded in a wearable artificial finger produced by a 3D printer, which was used as a finger in the UR5e robot (Figure 2C). Additionally, as shown in Figure 2A, a rigid metal appendage (length = 20 mm, diameter = 2 mm) was attached to the underside of the free end of the elastic metal sheet for impacting objects.

### 2.2 Selected spring steel material

**Figure 3:**
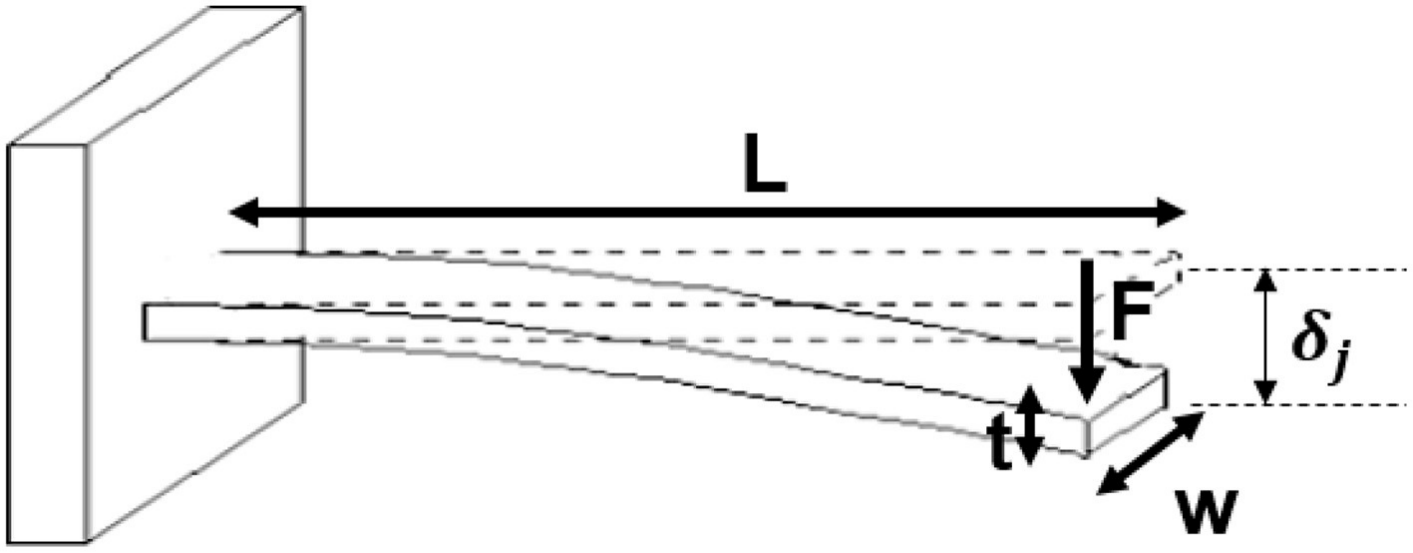
Dimensions of the cantilever beam.

Elastic deformation occurs when the applied stress does not exceed the yield strength of the material. In this case, when the force is removed, the spring steel returns to its original shape. It's crucial to use materials that stay within the elastic range after repeated compressions To ensure the applied stress remains below the material's yield strength, calculations determined an appropriate spring steel with a higher safety factor (60%) than the required working range, based on a force of 5.9 *N* and a deflection (δ_*j*_) of 16 mm. Therefore, the modulus of elasticity and the maximum stress developed are calculated according to the dimensions given in Figures 2A, 3 and Table 1 as follows:


(1) $$\begin{array}{l} E=\frac{FL^{3}}{3\delta_{j}I},\sigma_{max}=\frac{M_{max}c}{I} \end{array}$$

Where δ_*j*_ and *F* are the deflection and reaction force at the free end of the beam, respectively. *L* is the length of the cantilever beam, *E* is the modulus of elasticity of the material, and *I* is the moment of inertia of the beam's cross-section. Also, σ_*max*_ is yield strength of the material, *M*_*max*_ is the maximum bending moment and *c* is distance from the neutral axis to the farthest point of the section.

**Table 1:** Parameters selected in this study.

**Symbol**	**Definition**	**Value**
L	Length of cantilever beam	90 mm
w	Width of cantilever beam	25 mm
t	Thickness of cantilever beam	0.6 mm
C-D (δ_*j*_*max*__)	Maximum movements of metal appendage	10 mm
F	Maximum reaction force generated	3.7 N

Therefore, spring steel should be selected that meets *E* ≈ 199.8GPa and a yield strength (σ_*y*_) > σ_*max*_ = 300MPa. In this regard, we employed AISI 1074 carbon spring steel (*E* = 200GPa, yield strength = 385MPa) as the cantilever beam. Since the cantilever beam design was made with a higher safety factor, ensuring that the elastic deformation is maintained after multiple compressions.

### 2.3 Data acquisition

Signal type and quality significantly affect classification accuracy. Clear and relevant signals improve feature extraction, allowing models to better differentiate between classes. They also assist in identifying key features and boosting model generalization to unseen data (Asheghabadi et al., 2021). Thus, acquiring appropriate signals is crucial for reliable and efficient classification systems, affecting accuracy and performance. Therefore, according to Figure 1B, three types of data were recorded as follows:

#### 2.3.1 Damping vibration-force signals

The experimental platform was built with a UR5e 6-DOF collaborative robot and a two-finger gripper. The UR5e is a collaborative robot arm from the Universal Robots e-Series (Country: Denmark) that uses a motion planning algorithm offered by Robotics System Toolbox^*TM*^ to achieve joint space control, task space control, and waypoint tracking. Some important features include an error margin of ± 0.1 mm, a maximum payload of 5 kg, a reach of 850 mm, and a working temperature range of 0 to 50 °C.

The proposed finger was replaced with one of the gripper fingers (Figure 2C). Then, by positioning different objects on a table, the finger impacts the object vertically at a constant velocity from a distance of 5 cm, which causes vibration in the free end of the elastic metal sheet. To ensure robust data collection, four independent datasets were compiled over different time intervals, with each material being sampled 100 times per dataset. This systematic approach resulted in a comprehensive database comprising 400 samples for each material, thereby facilitating the evaluation of machine learning methodology. The accelerometer and load cell simultaneously record the damping vibration signal and damping force, respectively. The output of the load cell sensor was first entered into the 24-bit amplifier HX711. Then, the vibration and force signals were transferred to MATLAB software (version R2019b) through the data acquisition (DAQ), including an Arduino microcontroller (ATmega328P) with 16 MHz frequency and Bluetooth HC-05 with 2.4 GHz frequency. The transient signal, which the accelerometer records from a distance of 5 cm until the moment the metal appendage impacts the object, is excluded from the data set as it does not offer useful information and is constant across all data sets.

#### 2.3.2 Deflection of cantilever beam (δ_*j*_)

In addition to collecting vibration and force data, the cantilever beam deflection (δ_*j*_) value was recorded to facilitate data clustering. Initially, the robotic finger is positioned beside the surface without making contact. Subsequently, the finger moves to establish contact with the surface, and the metal appendage presses down until point D (Figure 2) touches the object's surface. This pressing action causes the cantilever beam's free end to move. This process was performed for all objects studied and the object's maximum reaction force on the metal protrusion was 3.7 *N*. The value of δ_*j*_ varies depending on the softness of different objects. After the measurement, the finger returns to the starting position at each step. Throughout the test, the total force applied to each material's surface and the distance from the finger to the surface remains constant, ensuring reliable and comparable data.

### 2.4 Feature extraction

Feature extraction is one of the important steps in the classification process where the raw data is transformed into meaningful features (Guyon and Elisseeff, 2006; Bahrami Moqadam et al., 2018). The feature vector should be simple, have low dimensions, and be able to create the highest correlation between the data of one class and the highest segregation among the classes (Moqadam et al., 2021b; Asheghabadi et al., 2022). The extracted features are provided as feed to the classification algorithms, and the accuracy and efficiency of the classification algorithms are directly affected by the quality and representation of the features. Improper features will affect the performance of classification algorithms and will suffer from overfitting. The feature vector can be considered as doing group work, in which, in addition to the fact that each component must perform well, they must also be able to perform the best together.

**Figure 4:**
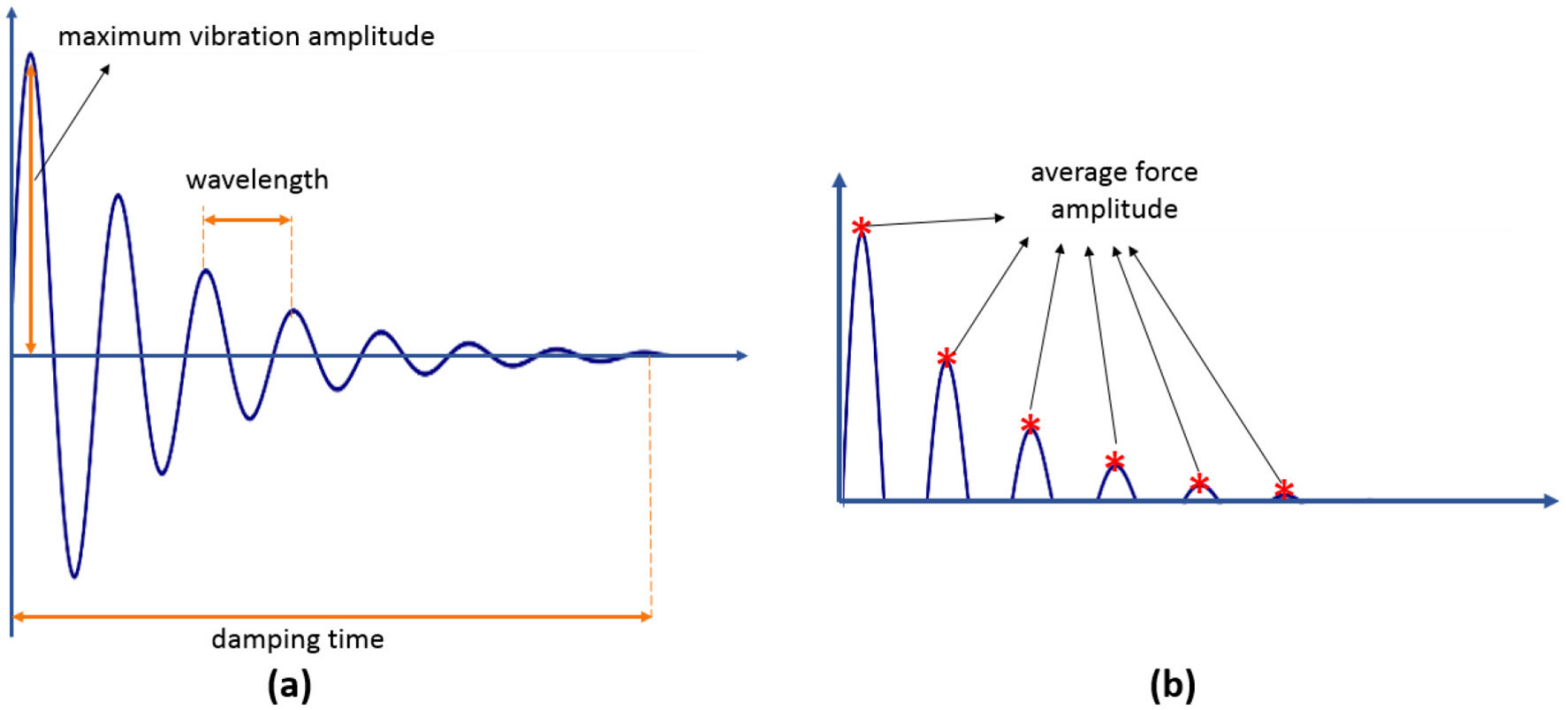
**(A)** Features extracted from damping vibration. **(B)** Features extracted from damping force.

After recording data (Figure 4), four features were extracted according to the steps shown in Figure 1C: the maximum vibration amplitude, the time it took to dampen, the wavelength, and the average force amplitude. Then, to validate the features and ensure their relevance and discriminative power for subsequent processing, we applied the analysis of variance (ANOVA) test and sequential forward feature selection (SFFS) with a significance level of 0.01.

Finally, the Davies–Bouldin Index (DBI) (Davies and Bouldin, 1979) was employed to measure the overlap of clusters based on the ratio of the sum of within-cluster scatter to between-cluster separation. Table 2 gives a description of each feature and its DBI values. Low DBI values indicate a high level of feature separability.

**Table 2:** Features and DBI value of features.

**Feature**	**Description**	**DBI**
Maximum vibration amplitude	Peak of signal	0.64
Damping time	The duration time of stopping the wave vibrations	0.58
Wavelength	The portion of a wave between two crests or troughs	0.45
Average force amplitude	Average signal peaks	0.53

### 2.5 Pattern recognition algorithms

Machines can make sense of complex information by using pattern recognition algorithms to identify patterns and regularities in data. This study applies three well-known artificial intelligence (AI) algorithms: linear discriminant analysis (LDA), multilayer perceptron (MLP), and support vector machine (SVM).

• LDA: is a supervised algorithm that uses linear feature combinations to optimally classify data and can handle feature correlation. Despite this, it may struggle in high-dimensional spaces.

• SVM: creates a hyperplane for high-accuracy classification in high-dimensional spaces with low memory requirements. Nevertheless, it struggles with noisy data, specifically when target classes coincide.

• MLP: is a widely used classifier that excels at nonlinear statistical modeling and can detect complicated relationships between variables. Nonetheless, its black-box character makes it less controllable. The multilayer perceptron (MLP) employed backpropagation and featured three hidden layers with 60, 55, and 35 neurons. Table 3 shows the sensitivity analysis of the MLP, which is significantly influenced by the number of neurones and layers.

**Table 3:** Effect of the number of neurons on the algorithm accuracy.

**Hidden layer**	**Number of neurons**	**Accuracy(%)**
2	[30, 30]	64.14
2	[35, 35]	67.73
2	[40, 40]	71.23
2	[45, 45]	74.13
2	[50, 50]	79.64
2	[55, 55]	77.16
2	[60, 60]	75.55
3	[45, 45, 45	84.74
3	[50, 50, 50]	85.88
3	[60, 60, 60]	83.13
3	[60, 55, 50]	84.65
**3**	**[60, 55, 35]**	**90.17**
4	[45, 45, 45, 45]	84.34
4	[55, 55, 55, 55]	82.22

The bold values indicate the best result within the number of layers and neurons.

The holdout method was used to divide the data into training (70%), and testing (30%) sets without any generation or augmentation of data. Each training dataset is labeled with material, allowing for the mapping of features to labels. The classifier's accuracy is assessed by testing it on materials with unknown labels.

## 3 Experiments and results

### 3.1 Objects

A material specifies the types of substances used and their combinations. Different compounds and molecular bonds create various mechanical properties in objects (Zhou et al., 2016), which can be used to distinguish materials. One of the important mechanical properties is the object's degree of softness or hardness, as well as its ability to dampen force. For instance, while steel and cast iron belong to the same family, their molecular structure causes differences in damping and vibrations.

**Figure 5:**
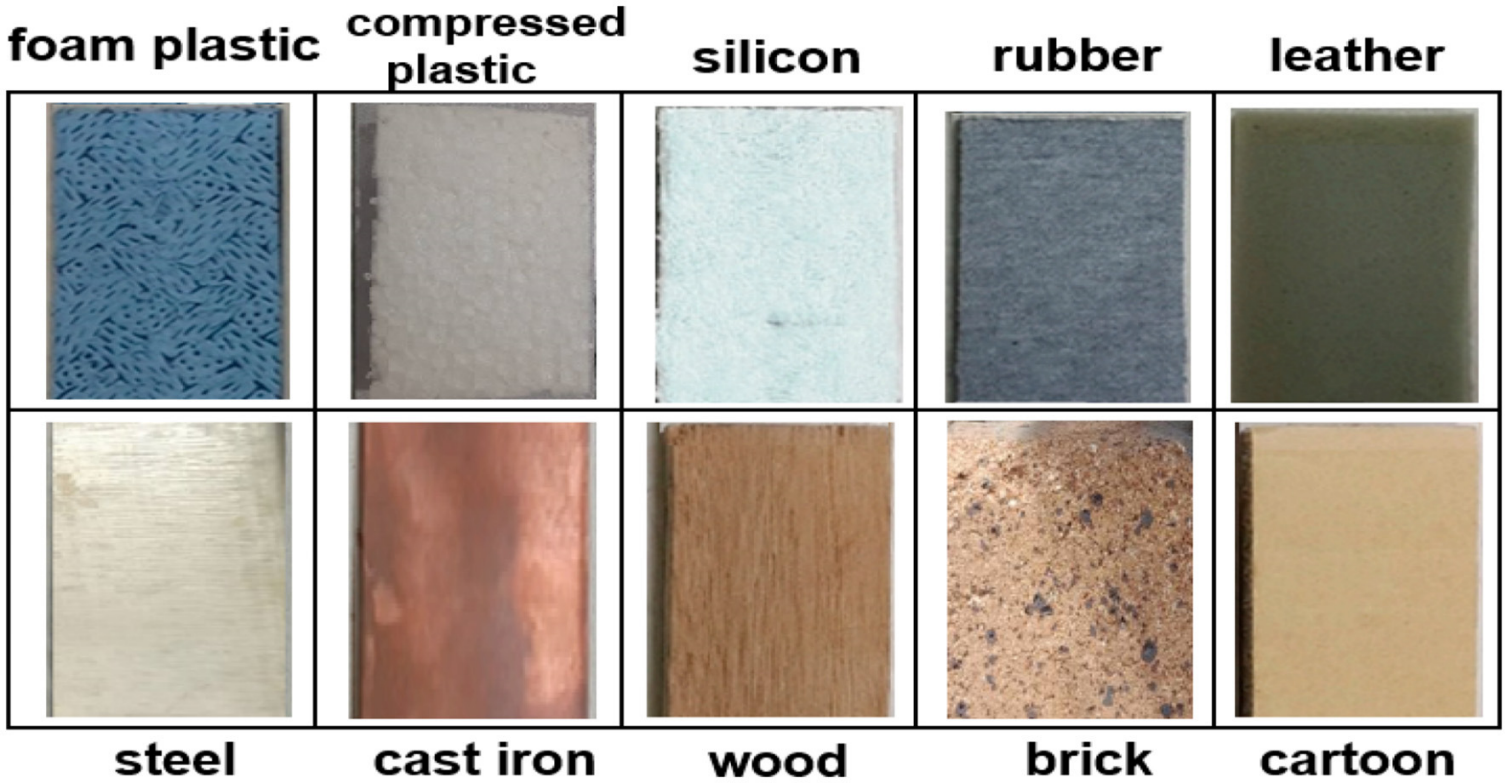
Object materials examined in this study.

To measure the materials, we prepared ten identical objects made from various materials, including 10 materials (foam plastic, compressed plastic, silicon, rubber, leather, steel, cast iron, wood, brick, cartoon) (Ma et al., 2023) (Figure 5).

In the standard samples, two different types were used for each family. For example, steel and cast iron are both members of the iron family. This factor was selected to investigate the performance of the presented method in the ability to differentiate almost similar materials.

### 3.2 Approach A: experiment for material detection

**Figure 6:**
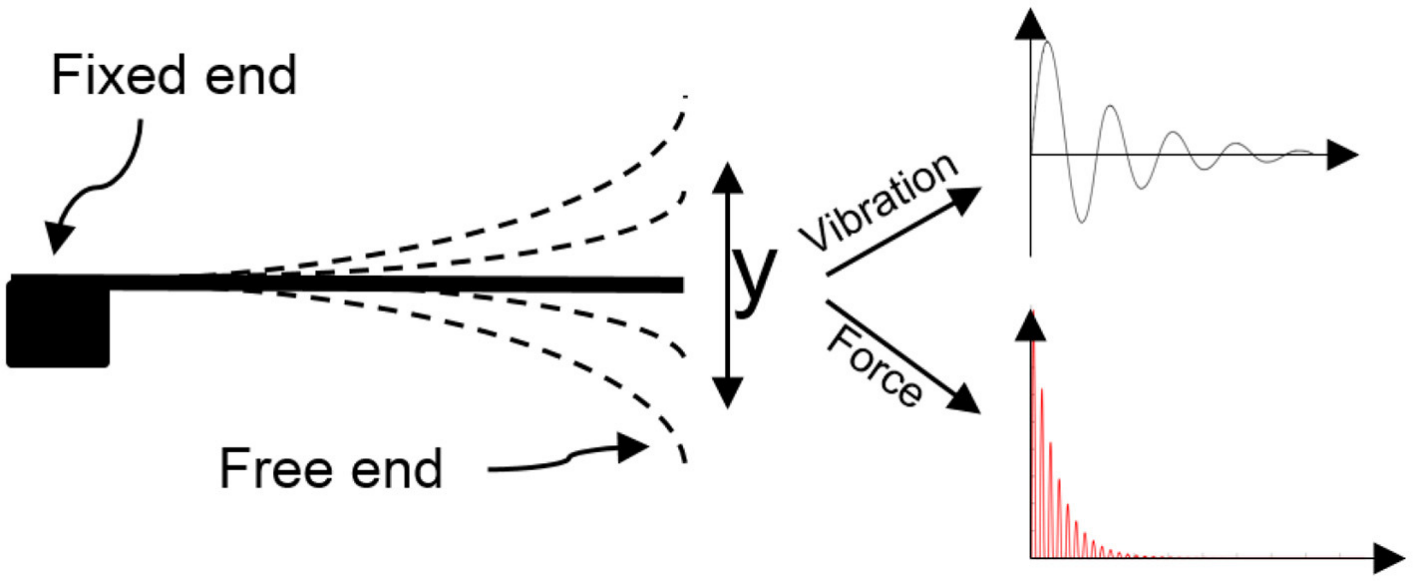
Working principle of the sensor for vibration-force measurement.

**Figure 7:**
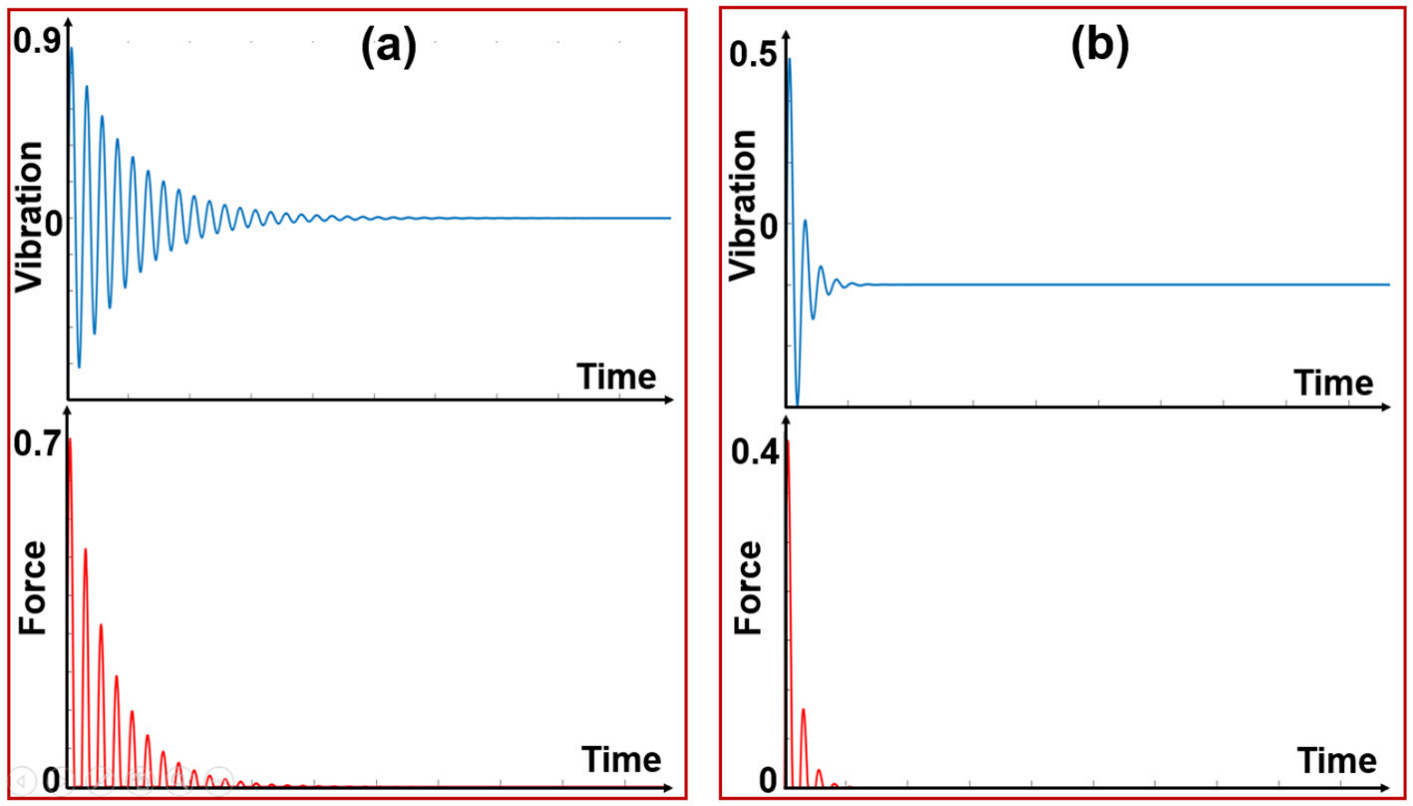
**(A)** Vibration-force signal from hard material, **(B)** Vibration-force signal from soft material.

After setting up the platform, data collection was done by the impact of the metal appendage on the object. In this way, a reaction force from the object is transmitted to the metal appendage that causes vibration on the free end of the elastic metal sheet, in which damping vibration and damping force are measured by the accelerometer and load cell, respectively. Figure 6 shows the principle of material detection in this study. The harder the object, the greater the reaction force it exerts on the metal appendage. Consequently, the free head of the elastic metal sheet vibrates more, leading to an increase in the amplitude and compression of the damping signal. Figure 7 shows the difference between the vibration-force damping signals between the two objects. Then the four mentioned features were extracted from the recorded signals of ten objects as a feed for the classification algorithms.

### 3.3 Approach B: experiment for material detection using object indentation as a clustering criterion

#### 3.3.1 Define δ_*j*_ as a clustering criterion

**Figure 8:**
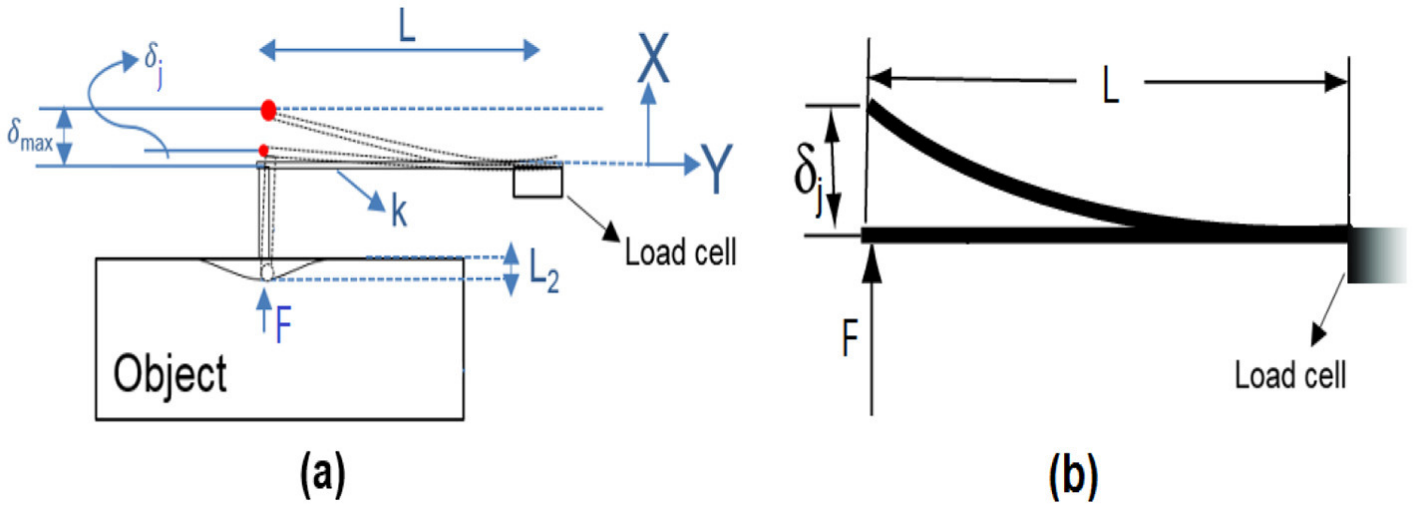
Working principle of the proposed sensor for δ_*j*_ measurement (L, length of the cantilever beam; δ_*max*_, maximum movements of metal appendage; δ_*j*_, cantilever beam deflection; F, reaction force; k, beam stiffness; L2, object indentation).

The metal appendage head compresses an object until point D (see Figure 2A) reaches the surface of the object. Then, the reaction force (*F*) from the object on the metal appendage of the cantilever beam is proportional to the beam deflection (δ_*j*_) according to Hooke's law (*F*=*k*_1_ δ_*j*_), measured by the load cell (Figure 8). In the elastic region, δ_*j*_ is calculated as follows:


(2) $$\begin{array}{l} F=k\delta_{j}\to \delta_{j}=\frac{F}{k}=\frac{FL^{3}}{3EI} \end{array}$$

Where *F* is the reaction force, *k* is the beam stiffness, δ_*j*_ is the beam deflection, *L* is the length of the cantilever beam, *E* is the modulus of elasticity of the material and *I* is the moment of inertia of the beam's cross-section. On the other hand, according to Figure 8A, the sum of the δ_*j*_ and the object indentation (*L*_2_) equals δ_*j*_*max*__, which is 10 mm; therefore, *L*_2_=10-δ_*j*_.

Finally, data is clustered into soft and hard groups based on δ_*j*_ values as follows:


(3) $$\begin{array}{l} \delta_{j}=\{\begin{array}{c} \text{if }0\leq \delta_{j}<10\text{ then object is soft}\\ \text{if }\delta_{j}=10\text{ mm then object is hard} \end{array} \end{array}$$

Hence, the maximum value of δ_*j*_ occurs when the object is hard and no indentation occurs. In soft objects, part of the force the metal appendage applies changes the object's shape. For instance, although wood is classified as a soft material in the Shore hardness criteria (Ma et al., 2023), in this study, it was classified as hard because δ_*j*_ = 10. It is worth noting that we utilized identical standard objects for this study. Furthermore, since our method involves single point contact, the data for clustering is received from one point only once. As a result, irregular deformations in this study do not affect the method's performance. Generally, the investigation of irregular deformations is beyond the scope of this paper.

#### 3.3.2 Improvement of the system performance based on δ_*j*_ value

**Figure 9:**
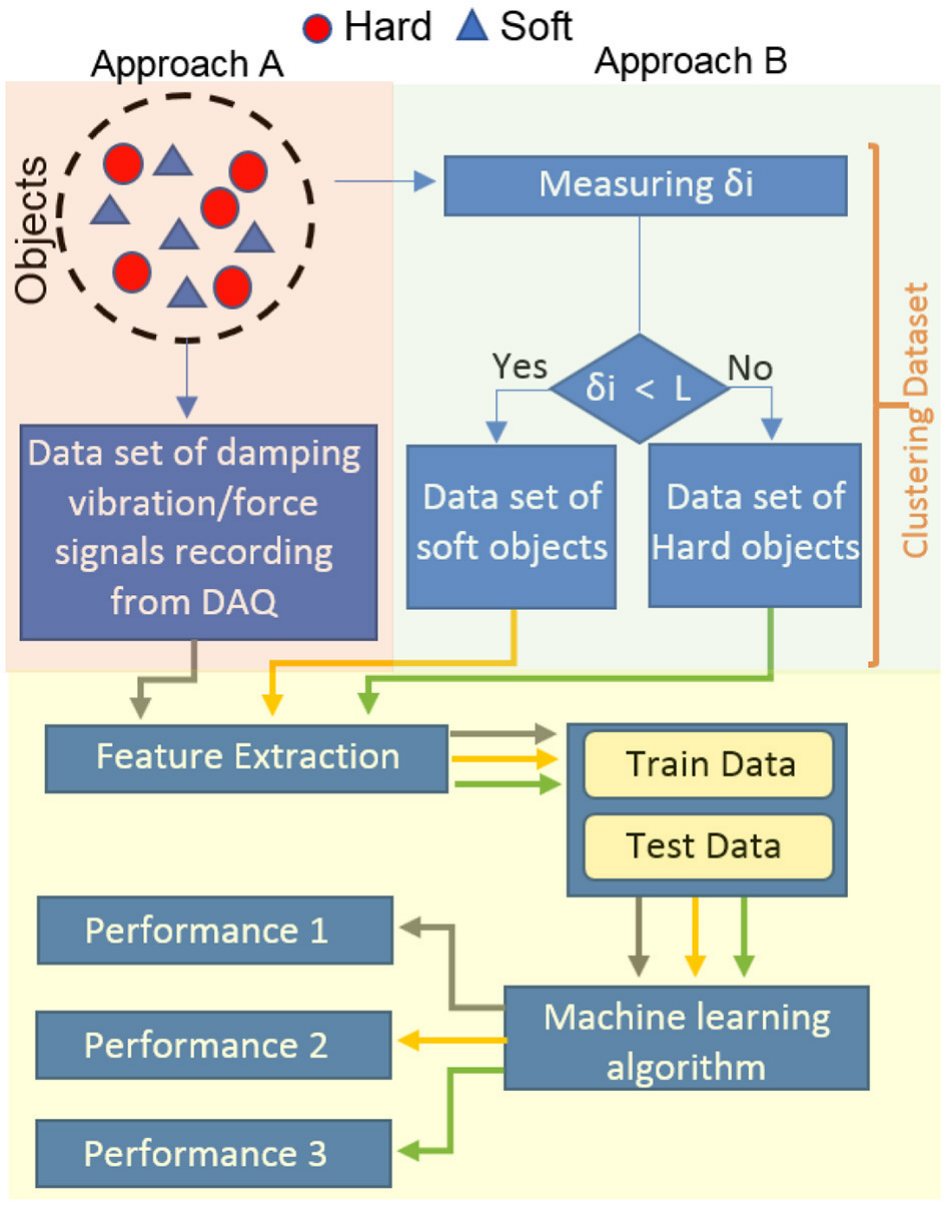
Comparison between approach *A* and *B*. Approach *A*: All data is input into the AI system, Approach *B*: Clustered data is input into the AI system.

Various factors, such as signal quality, noise, and interference, can affect the performance of pattern recognition algorithms. Reducing the input data simplifies the algorithm and decreases processor load (Asheghabadi et al., 2024). Unlike the regular approaches where all data is fed to AI, in this study the data is clustered into two categories based on δ_*j*_ values before being fed to the machine. According to Figure 9, clustering the vibration-force damping signals reduces the data input to the system. The percentage reduction of the dataset input to the machine can be expressed as follows:


(4) $$\begin{array}{l} R=\left(1-\frac{\left|u_{\left(i\right)}\right|}{\left|U_{\left(x\right)}\right|}\right)*100 \end{array}$$

Where *R* is the percentage of input data reduction, *u*_(*i*)_ is the number of selected data based on the δ_*j*_ values, *U*_(*x*)_ is the total number of data, and *i* is the index. This approach enhances the performance of approach *A* in the material classification of objects.

Finally, all algorithms were trained on a distinct training set and evaluated using a test set through the holdout method, which randomly selects training and testing data. This process is repeated ten times to acquire the average performance on the test sets.

**Figure 10:**
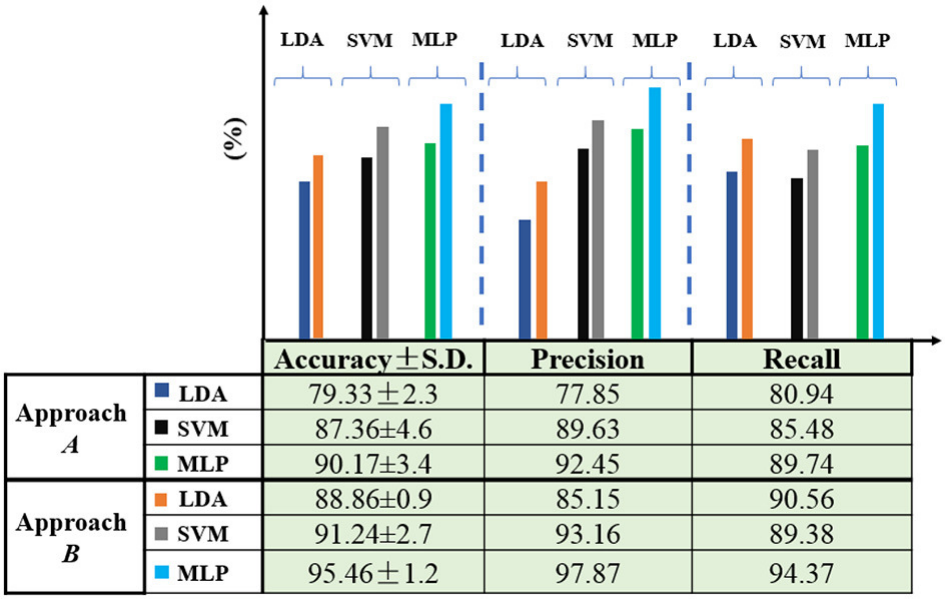
Comparison between approach *A* and *B* in three classifiers.

Figure 10 illustrates the system's performance in approaches *A* and *B*. In mode B, three classification criteria (accuracy, precision, and recall) showed improvement across the three classifiers. Additionally, the standard deviation (SD) decreased in mode *B*, indicating higher repeatability and reliability. In fact, material recognition was conducted in approach *B* with minimal variance within an inner class and maximum variance outside the class. Approach *A* only relied on the features extracted from the damping vibration-force signals resulting from the impact of the metal appendage on the objects. Approach *B* improved approach *A* by clustering data based on δ_*j*_ values. MLP achieved the highest accuracy at 95.46%.

Table 4 displays the confusion matrix for approach *B*. The confusion matrix offers a comprehensive view of the classifier's average performance. The majority of misclassifications occurred between materials that have similar properties. For instance, foam plastic and compressed plastic are both made of the same material. Similarly, steel and cast iron belong to the iron family, with differences in their production processes.

**Table 4:** Confusion matrix for material detection.

**Soft**							**Class**
Soft	*MATERIAL*	**F-P**	**C-P**	**Si**	**R**	**L**	F-P = Foam Plastic C-P = Compressed Plastic Si = Silicon R = Rubber L = Leather
	**F-P**	380	13	3	2	2	
	**C-P**	11	383	1	3	2	
	**Si**	2	2	390	3	3	
	**R**	2	2	5	388	4	
	**L**	1	1	2	5	391	
**Hard**							St = Steel C-I = Cast Iron W= Wood B = Brick C = Cartoon
Hard	*MATERIAL*	**St**	**C-I**	**W**	**B**	**C**	
	**St**	383	11	2	2	2	
	**C-I**	12	381	2	2	3	
	**W**	2	2	388	5	3	
	**B**	3	3	6	385	3	
	**C**	3	3	4	6	384	

The grey shade indicates the highest miss-classification (“C-P” and “C-I” had the highest classification error in soft and hard objects, respectively).

The results showed that most of the misclassifications by the algorithm are between objects made from the same material family. To investigate this issue, we trained the algorithm by grouping these materials as a single class. The following materials were grouped: (1) foam plastic and compressed plastic, (2) steel and cast iron. The total number of samples for each material is 400. To maintain consistent conditions for all objects, half of the data were randomly selected from the combined materials for training and testing (Approach *B*′). This led to a decrease in misclassifications and an overall improvement in classification accuracy, achieving 98.75%. Table 5 summarizes the evaluation indices in three modes among the classifiers.

**Table 5:** Evaluation metrics (%) of various classifiers for object material detection with two approaches.

**Approach**	**Feature**	**Classifier**	**Accuracy**	**Precision**	**Recall**	**SD**
**A**	**No clustering**	MLP	90.17	92.45	89.74	3.4
		SVM	87.36	89.63	85.48	4.6
		LDA	79.33	77.85	80.94	2.3
B	**Clustering with δ_*j*_**	MLP	95.46	97.87	94.37	1.2
		SVM	91.24	93.16	89.38	2.7
		LDA	88.86	85.15	90.56	0.9
**B′**	**Clustering with δ_*j*_ + combination**	MLP	98.75	99.17	100	0.8
		SVM	94.54	95.42	92.27	1.8
		LDA	91.55	90.67	93.15	0.5

The grey shade indicates the highest classification accuracy among other classifiers and methods.

## 4 Discussion

Robotics science aims to enhance and extend capabilities by performing tasks that may be beyond human abilities or require an exceptional level of precision and consistency without experiencing fatigue. To achieve this goal, researchers have developed Human-Computer Interaction (HCI) systems that facilitate restoring more natural behaviors. The HCI systems are designed to restore natural behaviors and movements, ultimately closing the gap between human and robotic functionality. A robotic system introduces significant noise into the system and distorts useful information, which can significantly affect the performance of pattern recognition algorithms. While previous studies concentrated on external object properties such as texture and roughness, our analysis centers on intrinsic object features to provide simple and meaningful information for classifiers. This study focuses on material detection with minimal sensor-object contact to minimize contact noise, thereby addressing the aforementioned problems in the learning process.

In this context, Table 6 compares our proposed material detection method with other studies' methods. Gandarias et al. (2017) employed two distinct AI methodologies to classify materials using pressure images obtained from high-resolution tactile sensors. The experimental outcomes revealed that the classification accuracy achieved using Speed-Up Robust Features (SURF) was 80%, whereas the classification accuracy obtained using Deep Convolutional Neural Networks (DCNN) was notably higher at 91.67%. Liu et al. (2018) introduced a tactile framework for identifying 10 different materials using an efficient feature extractor called the linear dynamic systems-based fuzzy c-means method (LDS-FCM). They then used the vector of locally aggregated descriptors (VLAD) method to derive the final features from the data. Their approach achieved an accuracy of 99%. Dai et al. (2022) created three tactile sensor designs with varying protrusions. They used a 2-axis Cartesian robot to receive signals by dragging the sensor on objects' surfaces. Ultimately, they could classify six materials based on their texture with 98.1% accuracy using the KNN algorithm and time-frequency domain features. Wang et al. (2022) utilized a tactile sensor with 16 small capacitors to capture tactile data from the robot's finger sliding across eight different types of fabric material. They were able to achieve 96% accuracy in classification by reducing the frequency domain features' dimensions using principal component analysis (PCA) and the k-nearest neighbor (KNN) algorithm. In previous studies, tactile methods have often used an array of sensors, which increases system noise and leads to more input data, necessitating more powerful and costly processors in addition to the problems mentioned earlier. Receiving signals from multiple sensors leads to signal interference and requires more complex solutions. Designing a system requires balancing the number of sensors, speed, user-friendliness, and noise levels. It's essential to find an equilibrium between model complexity and computational cost. Additionally, reducing channels can lower hardware costs and complexity, decrease controller processing time, and maintain high classification accuracy. This study introduced a novel sensor and reduced the number of sensors to one, enhancing classification accuracy by reducing the input dimensions. It's important to note that comparing different studies typically focuses on the sensor type and feature extraction algorithms, often neglecting other critical factors like object standardization, contact pressure, and exploratory measures. As a result, making valid comparisons between studies is not a straightforward task.

**Table 6:** Compares the presented method and other techniques.

**References**	**Method**	**Sensor**	**No. materials**	**Acc.(%)**
Gandarias et al. (2017)	SURF + k-mean + SVM/CNN + SVM	[28 × 50] tactile array	8	80/91.67
Liu et al. (2018)	LDS-FCM + VLAD	[3 × 8] tactile array	10	99
Dai et al. (2022)	KNN using time-frequency domain features	3 different superficial tactile sensors designs	6	98.1
Wang et al. (2022)	PCA + KNN	16 tactile sensors	8	96
This study	MLP using time domain features	1 impact sensor	10/8	95.46/98.75

Researchers have employed tactile and acoustic methods to enhance robots' capacity to identify object characteristics. Tactile methods are susceptible to factors such as unwanted vibrations and electrical signal interference, resulting in reduced accuracy and efficiency. Similarly, acoustic methods rely on microphones, which are not ideal for soft objects, and ambient noise can significantly degrade signal resolution. Both approaches demand substantial processing, leading to the creation of complex systems. However, literature has shown that contact control techniques can significantly increase the efficiency of robotic systems (Elguea-Aguinaco et al., 2023). Therefore, in this study, we utilized an integrated sensor employing the impact technique to minimize system noise and enable simultaneous measurement of two signals. The impact technique prevents excess noise transmission by establishing point contact, allowing the AI to focus on important information by reducing distracting signals. This approach reduces the system's processing load and enhances its detection accuracy and overall performance. By reducing the amount of processed data, the system can respond more quickly and accurately to environmental changes.

On the other hand, the feature space, defined by the type and number of features, significantly influences processor load (Asheghabadi et al., 2024). Therefore, minimizing the input data to the device streamlines the algorithm and lightens the processor load. Unlike conventional methods where all data is fed to the machine at once, in this study the data was clustered into two categories based on δ_*j*_ values before feeding into the AI. Figure 9 shows that clustering the vibration-force damping signals reduces the data input to the system. This approach enables the algorithm to utilize only relevant, high-quality data, eliminating complex feature extraction and unnecessary processing, thereby enhancing accuracy and processing speed. In particular, δ_*j*_ is a feature that is not included in the feature vector yet effectively divides the data and reduces the machine's workload. This method enhances the performance of approach *A* in object material classification by minimizing noise and interference, allowing the algorithm to identify more accurate patterns and improve classification accuracy. Compared to regular MFCC methods, this strategy reduced the dataset size by 50% and decreased feature extraction and classification times by 78% and 48%, respectively. This strategy significantly reduced the execution time. Ultimately, it optimizes processing time and boosts the efficiency of AI systems. Also, in previous studies, researchers classified materials by creating artificial textures on objects' surfaces, creating a predictable nature (Jamali et al., 2009; Edwards et al., 2008; Kim et al., 2005). Our work successfully classified materials based on intrinsic properties with the same natural surface. This approach also eliminated overfitting and enhanced system reproducibility. Features extracted from clustered data improved material recognition with minimal in-class variance, maximal out-of-class variance, and high repeatability. In this study, we exclusively used the δ_*j*_ value as the data clustering index (approach *B*). This index can be integrated with other methods, such as KNN, that employ Manhattan or Euclidean distance to create more boundaries for the data, dividing them into more categories.

The proposed method has some limitations as follows: (1) Sensitivity to the angle of impact when the sensor interacts with objects. Variations in the impact angle can affect vibration and force signal consistency, leading to inaccurate material classification. A possible solution is to use a mechanism that stabilizes the sensor's orientation or algorithms that normalize data to counteract angle variations, ensuring reliable signal acquisition. However, achieving this level of realism was beyond the scope of this study. (2) Reliance on a single-point contact for signal acquisition, while it reduces noise, might miss certain material characteristics that could be captured through a broader interaction. To address this, the sensor could be upgraded to include multiple points of contact or an array of miniaturized sensors. This would enable a more comprehensive collection of data across a larger surface area, providing a richer dataset that could improve the accuracy and robustness of the classification process without compromising the simplicity of the original setup. (3) The signals are often affected by uncertainties and inaccuracies, such as requiring preprocessing using tools based on fuzzy logic. To reduce computational load when dealing with large datasets, it is advisable to merge similar signals in a fuzzy manner (Versaci et al., 2020, 2022), thereby creating distinct fuzzy classes for each group and extracting a representative signal from each group.

It is expected that the proposed method will be used in other robot types, such as bionic hands (Moqadam et al., 2021a; Moqadam et al., 2022), where amputees face challenges interacting with the environment due to loss of touch sensation. In future work, in line with our previous studies, we intend to create sensory feedback in amputees (Moqadam et al., 2023a) with the help of myotome digits (Moqadam et al., 2023b) by embedding the proposed sensor in the hand prosthetic finger [which is controlled by electromyography signal (Bahrami Moqadam et al., 2018)] and making intelligent grasping to realize automated, robust, and accurate material detection.

## 5 Conclusion

This study presented an effective method for identifying objects' material by measuring damping vibration and force signals simultaneously. The direct proportionality between the measured force and the vibration of the elastic metal sheet led to the objects clustering into two groups, which reduced the input volume to the system and consequently improved classification accuracy. In this study, ten objects were classified using three different classifiers: MLP, SVM, and LDA, and the highest accuracy was 95.46% with MLP. Finally, the proposed method shows remarkable versatility, with potential applications not only in industrial automation but also in prosthetics, where the ability to recognize materials accurately can significantly enhance the interaction of robotic devices with their environment, making the research promising for future Implementations in various areas of advanced robotics.

## Data Availability

The original contributions presented in the study are included in the article, further inquiries can be directed to the corresponding authors.
